# How NHS Tayside dealt with the COVID-19 pandemic within Urgent Dental Care Centres: clinical staff views and experiences

**DOI:** 10.1038/s41415-022-4462-9

**Published:** 2022-07-19

**Authors:** Claire Scott, Brian Stevenson, Morag Curnow, Linda Young

**Affiliations:** 41415129811001 Dental Clinical Effectiveness, NHS Education for Scotland, Dundee, UK; 41415129811002 Dundee Dental Hospital, NHS Tayside, Dundee, UK; 41415129811003 Public Dental Health Service, NHS Tayside, Perth, UK

## Abstract

**Introduction** Dental staff are considered to be at increased risk of COVID-19 transmission, and national concerns about personal protective equipment (PPE) and staff safety have been widely reported. This study explores the views of staff working in Urgent Dental Care Centres (UDCCs) during the first COVID-19 lockdown.

**Aims ** To explore clinical staff views and experiences of PPE and personal safety while working in NHS Tayside's UDCCs during the initial months of the COVID-19 pandemic.

**Design** Cross-sectional questionnaire survey.

**Materials and methods ** A questionnaire was emailed to staff working within NHS Tayside UDCCs during the first lockdown. The questions related to PPE, working environment, personal safety and wellbeing. This paper focuses on PPE and personal safety.

**Results ** Of the 176 invited to participate, 116 completed the questionnaire. The majority confirmed that they always had access to appropriate PPE and few had concerns about personal safety.

**Discussion** Despite having worked in a high-risk environment throughout the lockdown, staff supporting the Tayside UDCCs felt safe and well-protected. This runs counter to widely reported anxieties about PPE and safety across the UK. Further investigation is required to understand this disparity.

**Conclusion ** The results indicate that PPE was adequate, accessible and staff felt protected.

## Key points

- Highlights that the strategies put in place by NHS Tayside ensured that staff felt safe while working within the UDCCs.
- Highlights that the strategies put in place by NHS Tayside ensured that staff felt well equipped with PPE while working within the UDCCs.
- Reports that these findings are contradictory to those widely reported in the media at the time.

## Introduction

The COVID-19 pandemic has had a global impact, with the first confirmed case reaching the UK in January 2020 and the first confirmed case in Scotland recorded in Tayside in March 2020. This coronavirus is highly infectious and transmitted through droplets and fomites.^1^ Dental team members are considered to be at increased risk of COVID-19 transmission due to working in close proximity to patients and the potential for spread through dental aerosol generating procedures (AGPs).^2^^,^^3^^,^^4^^,^^5^

On 17 March 2020, the chief dental officer in Scotland instructed that the delivery of all routine AGPs cease in NHS dental services. Short AGPs to address an urgent dental care need continued to be permitted for patients with no symptoms of COVID-19. COVID-symptomatic patients with an urgent dental care need could only be offered an AGP in a Health Board designated Urgent Dental Care centre (UDCC). A subsequent letter on 23 March 2020 announced that all face-to-face dentistry should cease from 'close of play' that day. From that date until 22 June 2020, all face-to-face urgent dental care was provided by regional Health Board UDCCs that had been set up by the Public Dental Service (PDS).

Additional guidance (*Management of acute dental problems during COVID-19 pandemic guidance*)^6^ was released in March 2020 by the Scottish Dental Clinical Effectiveness Programme in order to support initial telephone triage, triple-A management (advice, analgesia and antimicrobials) and onward referral to the UDCC where appropriate. Initial triage was carried out by the general dental practitioners (GDPs) for their own patients and by UDCC staff for unregistered patients. Patients who were deemed appropriate for face-to-face intervention were then referred on to the UDCC through a peer-to-peer structured conversation.

Within NHS Tayside, from 6 April 2020, volunteer GDPs undertook emergency clinics in conjunction with PDS and Hospital Dental Service (HDS) staff. Tayside is a large geographical area in Scotland with two major population centres (Dundee and Perth), numerous small towns and villages and extensive rural areas. The population is well-served with NHS, mixed and private dental practices, three PDS centres and a dental hospital and school in Dundee. The PDS and hospital sites were converted into UDCCs and staffed by primary and secondary care salaried dentists and support staff and volunteer GDPs. It was apparent that the UDCCs within NHS Tayside would have sufficient staff and space to see any dental problems at this time and it was agreed that same-day triaged referrals for fractured teeth, lost restorations (and other urgent dental care), even when presenting without pain, would be accepted and that this would continue as long as resources permitted. This method of working continued throughout and enabled almost 11,000 patients with dental problems to be seen within a three-month period across the four UDCCs in NHS Tayside.

The rationale behind the decision to work this way was that, in addition to having the resources and ability to meet the demand, it would be possible to intervene early and only once, rather than have multiple visits to pharmacies for potentially inappropriate medication which would in all likelihood still culminate in a visit to a UDCC.

Some clinical staff who worked in more than one Health Board area expressed concerns that access to clinical care was not consistent across the country and that seeing more patients was a potential risk to staff. These concerns were discussed at length within the senior management team and the individuals who had raised the issue. Consensus was that the patients' needs came first and that as many patients as could be safely seen should be, if resource allowed. The evidence supporting the safety of the systems in place within the UDCCs was reviewed and reiterated to the concerned staff members in writing and the wider staff at the daily huddles.

At the time, there were concerns across the UK about the availability, suitability and safety of personal protective equipment (PPE) for staff within dentistry.^7^^,^^8^ Level two PPE comprises of gloves, fluid-resistant mask, visor and disposable fluid-resistant apron. Level three replaces the simple surgical mask with a personally fitted FFP3 mask and added in a full body gown. NHS Tayside followed the appropriate levels of PPE that were advised at the time. In the initial stages of lockdown, choice of PPE was predicated on the perceived disease status of the patient.^9^ Latterly, it was transmission based, with level two PPE being worn for all non-AGPs and level three for AGPs. In NHS Tayside there had been a great deal of discussion and email traffic about what was safe and what was perceived to be safe. It was important that staff understood the different requirements for different patients or procedures and recognised that the PPE provided was appropriate. Therefore, extra care was taken to ensure this was explained to staff and that staff were comfortable with these decisions. Written queries were responded to in writing and daily huddles of staff addressed other concerns as they arose.

In order to evaluate whether the strategies taken by NHS Tayside UDCCs were effective in making their staff feel safe and well-equipped during the COVID-19 pandemic, a questionnaire was distributed to all clinical staff working within the UDCCs. This paper reports the results from the questionnaire and reflects on the impact of the strategies taken by NHS Tayside.

## Aims

To explore clinical staff views and experiences of PPE and personal safety while working in NHS Tayside's UDCCs during the initial months of the COVID-19 pandemic.

## Design

Cross-sectional questionnaire survey.

## Materials and methods

### Sample and recruitment

All clinical staff who had worked in any NHS Tayside UDCC were contacted by email and invited to complete a questionnaire on their experiences in the UDCCs during the COVID-19 pandemic. One reminder was sent close to the closing date.

### Questionnaire development

The questionnaire was developed with the input of the acting clinical director of Dundee Dental Hospital, clinical dental director of the PDS in NHS Tayside and members of NHS Education for Scotland's Dental Clinical Effectiveness team. It included questions relating to PPE, the working environment, staffing, workload and work-related pressures. These were answered through a series of tick box options, Likert scale and free-text-answer questions. This paper focuses on the results of the questions relating to PPE and personal safety.

### Data collection

Data were collected using an online questionnaire hosted on Questback, an online survey feedback tool which is often used to help improve services and resources. Participants were sent the questionnaire link via email.

On clicking the invitation link, potential participants were directed to an introduction page which provided information about the study aims, data use and purpose, confirmed that responses would be anonymous and that when published would not be identifiable. Consent to participate was then implied by continuing to the start of the questionnaire and completing the questions.

### Data analysis

Quantitative data were analysed using IBM SPSS Statistics v24. For each section in the questionnaire, descriptive statistics were used to calculate response frequencies and means as appropriate. Mann-Whitney U tests were used to compare responses by professional role where appropriate. Due to multiple testing, the *a priori* criterion for statistical significance was set conservatively at p <0.01.

### Governance

The study was assessed using the NHS Health Research Authority defining research online decision-making tool. This tool confirmed that the study would not be considered research by the NHS and that NHS Research Ethics Committee review was not required. The study was governed in accordance with NHS Education for Scotland's guidance for the conduct of evaluation studies and institutional ethical review was also not required.

All data collected was anonymous and was managed according to NHS Education for Scotland's information governance policies and procedures.

## Results

### Demographics

The questionnaire was sent to approximately 176 clinical staff working in NHS Tayside UDCCs. A total of 116 staff completed the questionnaire, a response rate of 66%. Respondents comprised of dentists (68%), dental nurses (25%), hygienists (3%), therapists (1%) and other (4%). The number of years practising ranged from 2-46 years, with an average of 19 years (standard deviation = 11.5). Overall, 77% of participants were women, 16% were men and 7% chose not to say.

### Working with non-COVID-19 and COVID-19-positive or isolating patients (non-AGPs)

In total, 96% of participants (n = 111) stated that they worked with non-COVID-19 patients providing or assisting with non-AGPs and 24% (n = 28) stated that they worked with COVID-19-positive or isolating patients, providing or assisting with non-AGPs.

**Fig. 1:**
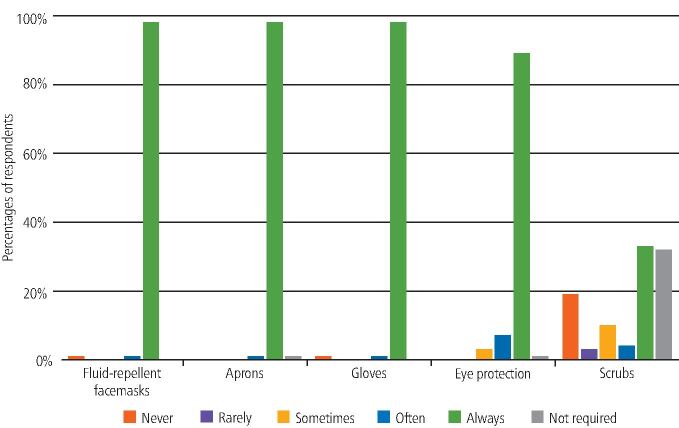
Access to PPE within the UDCC (non-COVID-19 patients)

**Fig. 2:**
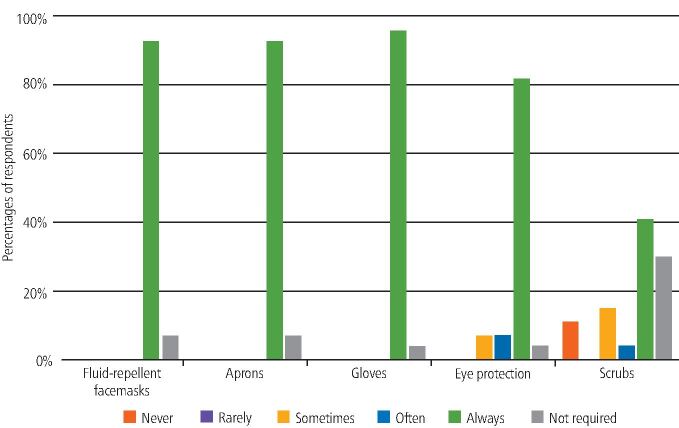
Access to PPE within the UDCC (COVID-19-positive patients)

Participants were asked if they had easy access to various types of PPE when working with non-COVID-19 and COVID-19-positive or isolating patients. The majority selected 'always' for most types of PPE; however, the response for scrubs was more varied (Figures 1 and 2).

When asked if they felt pressure to work with non-COVID-19 patients without the designated PPE, 94% of participants selected 'never'. Similarly, when working with COVID-19-positive or isolating patients, 82% of participants selected 'never'.

**Fig. 3:**
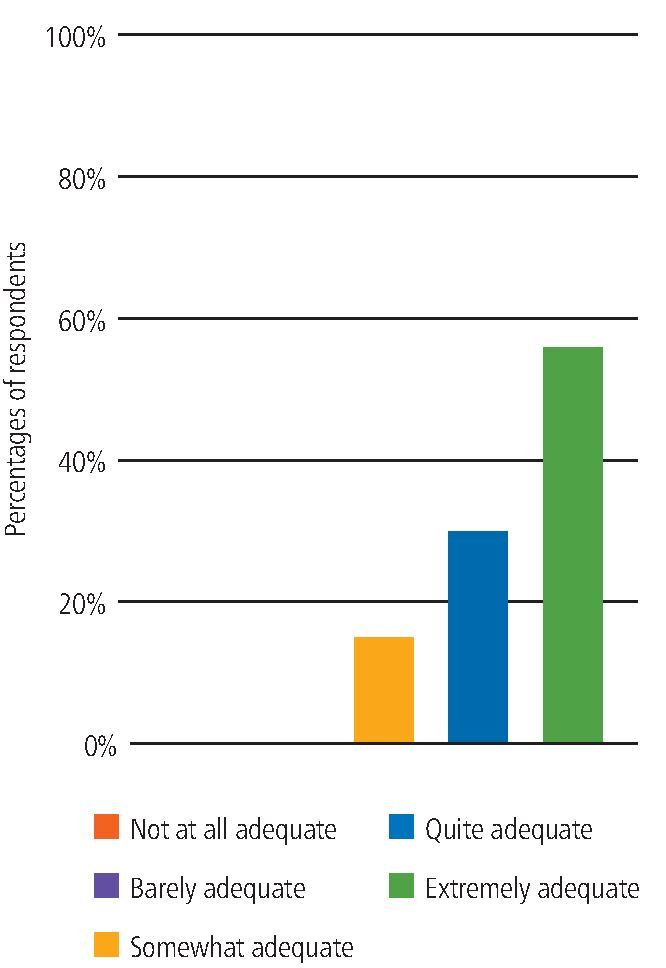
Adequate PPE when working with COVID-19-positive or isolating patients

Overall, 92% of participants working with non-COVID-19 patients rated the adequacy of the designated PPE as either 'quite adequate' or 'extremely adequate' and 85% of participants working with COVID-19-positive or isolating patients (non-AGP) rated the adequacy of the designated PPE as either 'quite adequate' or 'extremely adequate' (Fig. 3).

In terms of how protected from COVID-19 participants felt when working with non-COVID-19 patients in the UDCCs (non-AGP), 78% felt fully or highly protected, 2% 'moderately protected' and 1% 'barely protected'. None felt 'not at all protected'.

**Fig. 4:**
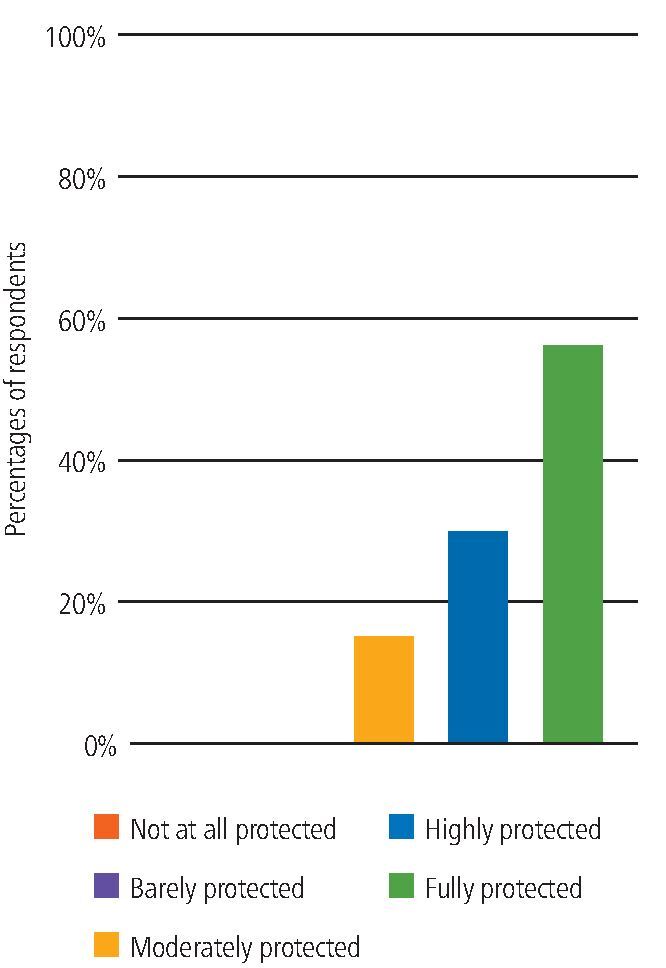
Feeling protected when working with COVID-19-positive or isolating patients

When working with COVID-19-positive or isolating patients in the UDCCs (non-AGP), the majority of participants (85%) felt fully or highly protected (Fig. 4).

### Delivering or assisting with AGPs in the UDCCs

Of the participants, 47% (n = 54) stated that they delivered or assisted with AGPs within the UDCCs and therefore completed the AGP section of the questionnaire.

**Fig. 5:**
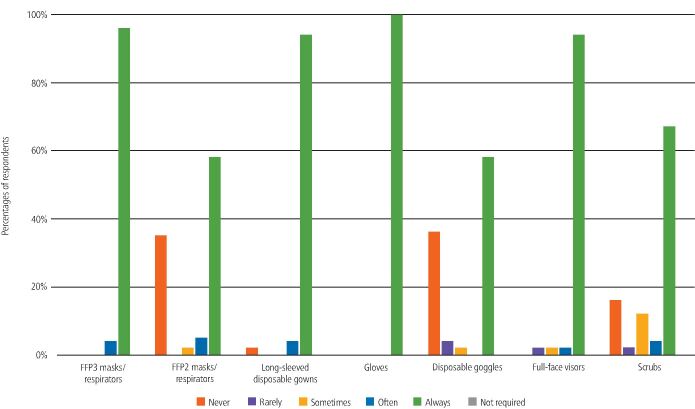
Access to PPE for AGPs within the UDCC

These participants were asked if they had easy access to various types of PPE when delivering or assisting with AGPs in the UDCCs. The majority of participants selected 'always' for each type of PPE (Fig. 5).

**Fig. 6:**
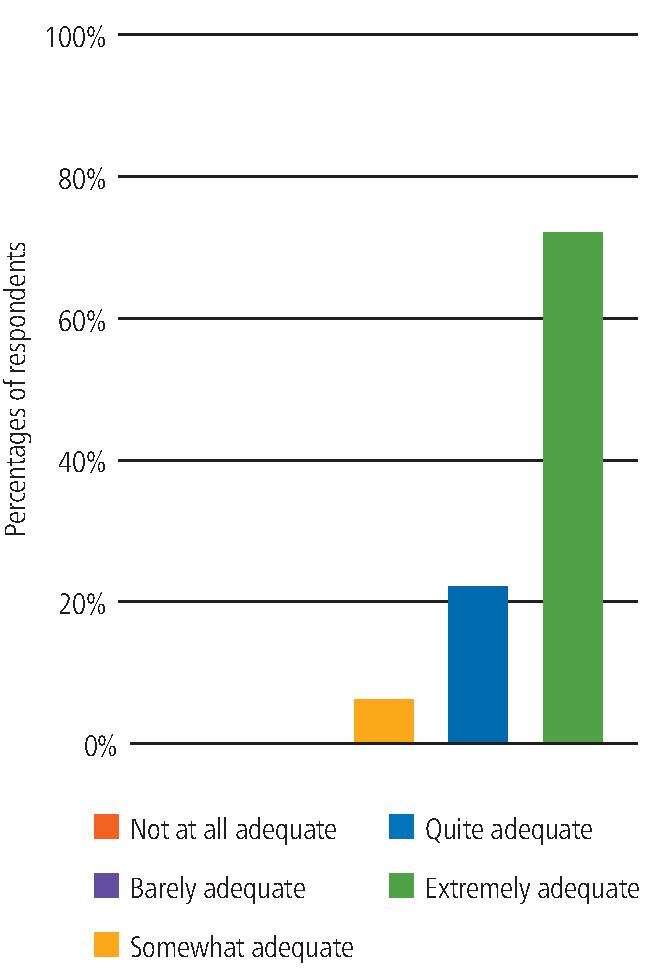
Adequate PPE provided when delivering or assisting with an AGP

When asked if they ever felt pressured to deliver or assist with an AGP without the designated PPE within the UDCC, 93% of participants selected 'never'. Also, 94% of participants involved in delivering or assisting with AGPs scored the designated PPE as either 'quite adequate' or 'extremely adequate' (Fig. 6).

**Fig. 7:**
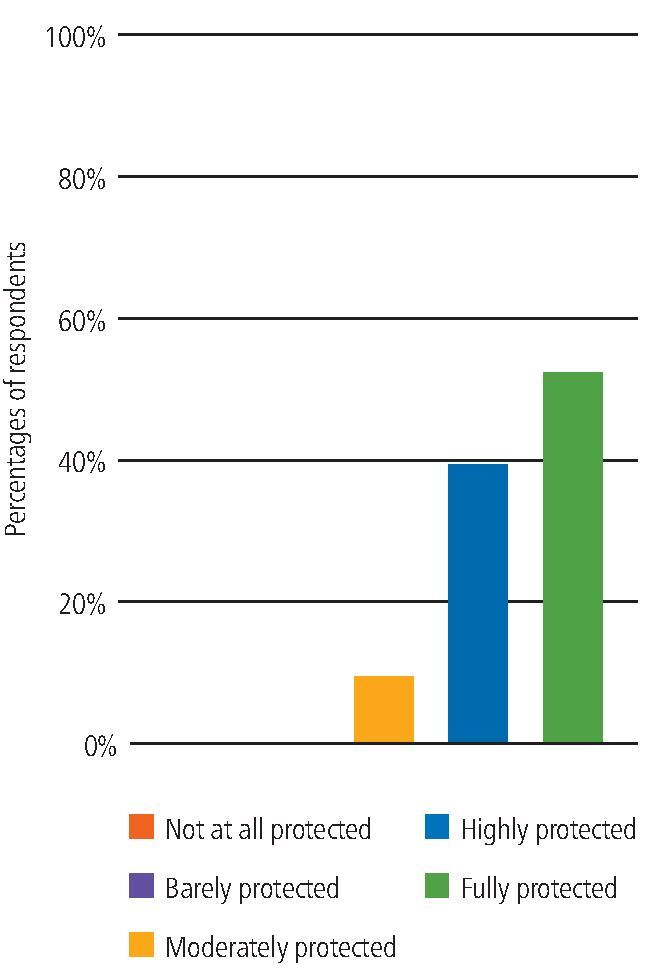
Feeling protected when delivering or assisting with an AGP

When asked how protected from COVID-19 participants felt when delivering or assisting with an AGP in the UDCCs, the majority (91%) of participants felt either fully or highly protected (Fig. 7).

When asked if they had reported or spoken out about any concerns they had while working in the UDCC regarding specific COVID-19-related issues, the majority reported they had 'no concerns' (PPE [78%], staffing [74%], personal situation [66%] and COVID-19 testing [81%]). Of the 26 participants who had a concern about PPE, one-third had not spoken out about them. The most common reason given for not speaking out was 'do not believe any action will be taken'.

**Fig. 8:**
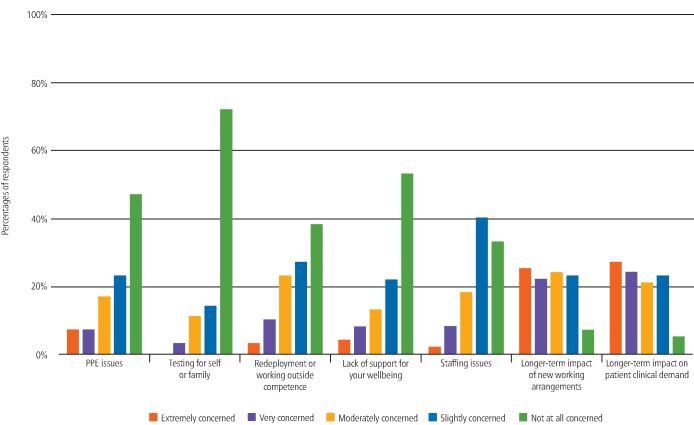
Rating level of concern regarding various issues relating to the COVID-19 pandemic

Finally, participants were asked to rate how concerned they are about certain areas regarding the pandemic. The highest levels of concern were about the long-term impact of working arrangements and patients' clinical demand. The issues that participants had the least concern about were testing for themselves/family and support for their wellbeing (Fig. 8).

**Fig. 9:**
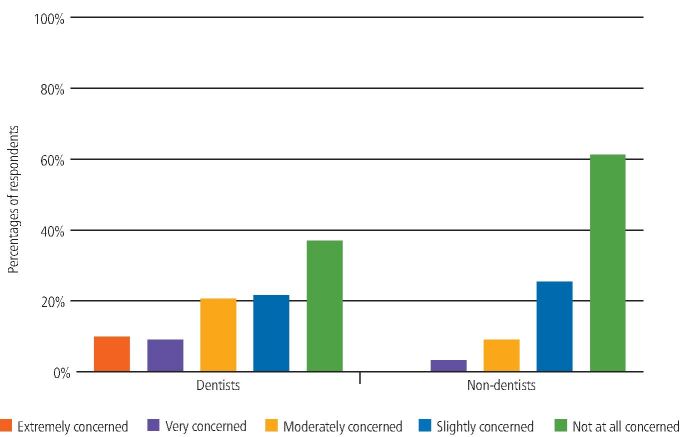
Dentists' and non-dentists' rating of level of concern regarding PPE relating to the COVID-19 pandemic

When looking at the data broken down by professional role, for 'PPE issues', 63% of non-dentists selected 'not at all concerned' compared to 38% of dentists (Fig. 9). Comparison of the responses from dentists and non-dentists found a significant difference (U = 884, p = 0.002).

## Discussion

The results of the questionnaire indicate that overall staff found PPE easily accessible and that the level of PPE supplied was considered 'adequate'. It also showed that staff did not feel pressured to work without the designated PPE and recognised that the designated PPE was the correct PPE. In terms of staff safety, they appeared to feel generally well-protected from COVID-19 while working within NHS Tayside UDCCs. These findings contradict the fears reported in the media^10^ surrounding the availability, suitability and safety of PPE and therefore the safety of staff within dentistry in the UK. This suggests either that Tayside dental services were unique in their provision of PPE and communication with staff, or previous media articles did not explore and report the whole story. Previous qualitative research conducted in England^11^^,^^12^ found through interviews that staff in their UDCCs felt uncertain over their safety^11^ and had concerns about PPE and safety,^12^ which again differ from the findings from this study. It was also found that non-dentists were less concerned about PPE than dentists.

This study was carried out to help evaluate the impact of NHS Tayside strategies on staff experiences in UDCCs. It was key to establish that staff had appropriate PPE, that they understood what PPE was appropriate in different roles and settings and trusted their employers to keep them safe. Although a small number of staff had reservations about raising concerns with their managers, it is encouraging that more than three-quarters of those with a concern had no reservations in raising these.

The senior leadership team, comprising of senior dental officers, the leads for PDS and HDS, the dental practice advisor and the chair of the Area Dental Advisory Committee, met regularly online from mid-March. Collective decisions made were enacted throughout the UDCCs and with the continuing support of dentists in independent practice. A challenge inherent in managing clinical need in a novel virus pandemic is the lack of baseline knowledge and the need to change processes and protocols as evidence emerges. Treating each change as an improvement rather than as the correction of a preceding error and communicating constantly with the frontline providers of the service was essential in building and maintaining team spirit.

By having adequate staff, including volunteers from independent practice and the space available to them, NHS Tayside was able to make the decision to see same day triage referrals and therefore were able to see a large number of patients in a short time, making their service more efficient and user-friendly.

NHS Tayside was also confident they had the adequate level of PPE in order to keep their staff safe and shared this confidence with staff at all levels.

More research is required to establish how justifiable staff anxieties can be allayed in future healthcare crises.

### Limitations

This was a self-selecting sample of all clinical staff who worked in any Tayside UDCC at any time in the first lockdown. However, the rate of return was high at 66% compared to reported averages for organisational and dental surveys at 53% and 63%, respectively^13^^,^^14^ and the demographics of the responders were consistent with those of the staff. Also, due to the nature of study design, it relies on self-reporting through a questionnaire which is thought to be open to self-reporting bias.^15^ However, questionnaires are also thought to be valuable for gathering data on opinion and experience^13^ and therefore the most appropriate design for this study.

## Conclusion

The findings of this questionnaire indicate that the strategies put in place by NHS Tayside have ensured that staff felt well-equipped and safe while working within the UDCCs. A recent study in Norway confirmed that where staff felt well-protected, they were more secure and less fearful.^16^ Open communication, availability, and listening, liaising and cooperating with staff at all levels is key. Management need to model good communication, flexibility and adaptability to rapidly changing circumstances to support and encourage staff to share learning and enable change.

The results of this questionnaire will help to support NHS Tayside to fully evaluate its response to the COVID-19 pandemic and will inform further development and support for staff during this pandemic and in the future.

## References

[1] Cao W, Li T. COVID-19: towards understanding of pathogenesis.* Cell Res *2020; **30:** 367-369. 10.1038/s41422-020-0327-4 PMC7186532 32346073

[2] Shields A M, Faustini S E, Kristunas C A *et al.* COVID-19: Seroprevalence and Vaccine Responses in UK Dental Care Professionals. *J Dent Res *2021; **100:** 1220-1227. 10.1177/00220345211020270 PMC8461044 34077690

[3] Meng L, Hua F, Bian Z. Coronavirus Disease 2019 (COVID-19): Emerging and Future Challenges for Dental and Oral Medicine. *J Dent Res* 2020; **99:** 481-487. 10.1177/0022034520914246 PMC7140973 32162995

[4] Pedrosa P B S, Cardoso T A O. Viral infections in workers in hospital and research laboratory settings: a comparative review of infection modes and respective biosafety aspects. *Int J Infect Dis *2011; DOI: 10.1016/j.ijid.2011.03.005. 10.1016/j.ijid.2011.03.005 PMC7110847 21497126

[5] Van Doremalen N, Bushmaker T, Morris D H *et al.* Aerosol and Surface Stability of SARS-CoV-2 as Compared with SARS-CoV-1. *New Engl J Med *2020; **382:** 1564-1567. 10.1056/NEJMc2004973 PMC7121658 32182409

[6] Scottish Dental Clinical Effectiveness Programme. Management of Acute Dental Problems During COVID-19 Pandemic Guidance. 2020. Available at https://www.sdcep.org.uk/published-guidance/acute-dental-problems-covid-19/ (accessed June 2022).

[7] British Dental Association. Dentists: PPE shortages leaving staff at risk and urgent care system in jeopardy. 2020. Available at https://bda.org/news-centre/press-releases/Pages/PPE-shortages-leaving-staff-at-risk-and-urgent-care-system-in-jeopardy-.aspx (accessed June 2022).

[8] Edgington T. Coronavirus: Dentists facing 'critical shortage of kit'. *BBC* (London) 2020 April 21.

[9] Health Protection Scotland. Annex 1: Infection Prevention and Control in Urgent Dental Care Settings during the period of COVID-19. 2020. Available at https://www.scottishdental.org/wp-content/uploads/2020/04/HPS-Urgent-Dental-Care-COVID-19-April-11.pdf (accessed June 2022).

[10] Peakin W. 'Don't use Scottish Government supplied PPE,' BDA advises. *Scottish Dental Magazine *(Paisley) 2020 August 28.

[11] Plessas A, Paisi M, Baines R *et al.* Frontline experiences and perceptions of Urgent Dental Care centre staff in England during the COVID-19 pandemic: a qualitative study. *Br Dent J* 2021; DOI: 10.1038/s41415-021-3375-3. 10.1038/s41415-021-3375-3 PMC8420962 34489544

[12] Sandhu B K, Blanchard J R, Koshal S. COVID-19 - the impact on wellbeing of the dental team in a secondary care urgent dental hub. *Br Dent J *2021; DOI: 10.1038/s41415-021-3317-0. 10.1038/s41415-021-3317-0 PMC8390019 34446841

[13] Tan R T, Burke F J T. Response rates to questionnaires mailed to dentists. A review of 77 publications.* Int Dent J* 1997; **47:** 349-354.

[14] Baruch Y, Holtom B C. Survey response rate levels and trends in organisational research. *Hum Relations *2008; **61:** 1139-1160.

[15] Althubaiti A. Information bias in health research: definition, pitfalls, and adjustment methods. *J Multidiscip Healthc* 2016; **9:** 211-217. 10.2147/JMDH.S104807 PMC4862344 27217764

[16] Uhlen M M, Ansteinsson V E, Stangvaltaite-Mouhat L *et al. *Psychological impact of the COVID-19 pandemic on dental health personnel in Norway. *BMC Health Serv Res* 2021; **21:** 420. 10.1186/s12913-021-06443-y PMC8092364 33941194

